# The Uniqueness and Ordinariness of Cancer Origin and Pathogenesis: New Epidemiological, Clinical and Preventive Perspectives

**DOI:** 10.4021/jocmr2009.02.1223

**Published:** 2009-03-24

**Authors:** Sergey N. Rumyantsev

**Affiliations:** Andent Inc., 1000 North Avenue, Waukegan, IL 60085, USA Email:rumyan1@yahoo.com

## Abstract

**Background:**

The article is devoted to a try to reconsider a main paradigm of contemporary oncology, the hypothesis of cancer metastasis.

**Methods:**

The investigation was based on the reassessment of well known data about cancer epidemiology and clinical manifestations from the viewpoint of recent all-pathological, immunological, genetic and evolutionary discoveries.

**Results:**

The potentially cancerous cell clones settled a human body as a result of crossbreeding between persons with partially different genomes. The clones appeared in the body before postnatal ontogenesis and for many decades exist in it as multiple but smallest stochastically distributed cell populations. But at relevant time of individual life (mainly after 40 years of age), according to own specific programs of the clone ontogenesis, it begin to multiple uncontrolled thus initiating the cancerous growth being unregulated by usual cyto-ecological agents owned by affected body. Its cells come into sight as constitutionally immune to normal regulators of cell reproduction and tissue growth.

**Conclusions:**

The new notion provides a framework for understanding the epidemic nature of cancer and its rising incidence both in the developed world and in developing countries. It forces also to reassess the means and methods of cancer healing. What is more, it accents the possibility of genetic methods for the prevention of epidemic spread of the disease.

**Keywords:**

Cancer epidemiology; Cancer pathogenesis; Genetics of cancer origin; Genetic immunity; Molecular physiological regulation

## Introduction

Cancer is the second most common cause of death in many developed countries. In the USA, for instance, cancer is accounted for 1 of every 4 deaths. It is exceeded today only by cardiovascular diseases. What is more, the cancer incidence continues to arise. Despite considerable epidemiological, immunological, genetic, surgical and pharmacological efforts, cancer continues to increase its toll in human death. Beside, the efficacy of means recommended for cancer prevention and treatment is very low.

The knowledge of origin and initial causes of the disease is far away of satisfactory clearness but contains some questionable assertions including both the compulsory existence of maternal (primary) tumor and inevitable spread (metastasis) of cancer cells from maternal tumor to form new (daughter's) tumors in distant locations in the body. Distantsite metastases are considered the leading cause of cancerassociated mortality.

It is thought the cancer cells spread mainly through the bloodstream or the lymph system. But in reality only the end point of the process--macroscopic metastases is observed. The details of the metastatic process remain hypothesized and mysterious [1]. The present article is devoted to a try to initiate a revision of the two questionable assertions of contemporary oncology basing on well known data about the cancer manifestations and course [2] in comparison with the data of recent all-pathological, epidemiological, immunological and genetic discoveries.

The preliminary hypothesis was that potentially cancerous cell clones appear in a body as a result of interbreeding between persons with partially different genomes. The clones arise in the body during yearly stage of ontogenesis and for many decades exist in it as multiple but smallest stochastically distributed cell populations in full concordance with general rules of the regulation of cell reproduction and tissues' growth. But at relevant time of individual age (mainly after 40 years of life), according probably to own specific program of ontogenesis, the cells of relevant clone begin its malignant development being constitutionally immune to normal cyto-ecological regulators.

## Materials and Methods

The investigation was based on the reassessment of well known data about cancer epidemiology and clinical manifestations from the viewpoint of recent all-pathological, immunological, genetic and evolutionary discoveries. The first step in this new contribution to cancer descent and epidemic spread has been initially supported by the Science Advisory Board publication on www.scienceboard.net [3].

## Results

The investigation included, first of all, the recently discovered all-pathological prerequisites (a), for subsequent reassessment of the unique and ordinary features of cancer disease (b), and the data of recent discoveries on genetic immunity of cells against molecular cyto-physiological regulators(c). The preliminary results allowed proposing a model of cancer molecular pathogenesis (d) and explaining the specificity of genetic predilection to cancer (e).

### (a) All-pathological prerequisites

Beside with very specific and extraordinary various manifestations any disease displays also a set of universal signs that are characteristic of any other disease too. Each of these universal features illustrates all-pathological phenomenon of heterozygous mosaicism created by genetic admixture. The mosaicism is the condition in which the phenomenon of heterozygosity reveals genetically determined variations in the locations, sizes and other pathological manifestations of any disease on the level of species, populations, organs, tissues, cells and molecular make-up of individual bodies [4].

The mosaic composition of living beings arises as a result of heterozygous self-reproduction. It reveals itself especially intensively in any form of both infectious and noninfectious pathology determining individual differences in the manifestations, course and severity of diseases. This kind of biodiversity arises as a result of sexual self-reproduction resulted in hybridization between two genetically different organisms: one of them is constitutionally immune to relevant ecological or physiological agent whereas its mating partner is constitutionally sensitive to it.

The hybridization forms inevitably either this or that grade of heterozygosity, which is resulted in the coexistence in the body at least of two active allelomorphic genes and of two allelic cell clones. Both of these alleles function dominantly. As a result, the cell populations of a descendant's body are formed under control of two codominant allelomorphic genes. The heterozygous individual shows both alleles expressed equally but in separated sizes and locations around the body [4].

### (b) The uniqueness and ordinariness of cancer

Any disease is characterized by a set of universal allpathological signs, features. This set includes at least a dozen of intrinsic features: a) different incidence of the disease among the races and ethnic groups, b) increased prevalence of diseases in developed and civilizing countries, c) genetic predilection to the disease, d) age differences in the incidence of disease, e) stochastic distribution of individual cases amongst a population, f) individual variations in constitutional (genetic) predilection to the disease, g) the mosaicism of affections, i.e. intra-individual diversity both in the predilection of different parts of a tissue and in the quantity and sizes of affections, h) stochastic distribution of affections amongst a body, and i) molecular bases of genomic and cellular pathogenesis k) the identity of involved cells in any locations of specific affections around the body [4]. Each of these well known universal signs is belong to any form of cancer. Beside, any of them contains either this or that contradictions to the revised paradigm.

In contrast, unlike to other kinds of diseases the appearance of malignancy is very uniquely, i.e. cancer come in sight when the dividing, growth and differentiation of some cells in some parts of a body' tissue are uncontrolled and crazed. It occurs when some cells of a tissue are physiologically abnormal and divide without control or order. The disturbance is determined by constitutional (genetic) immunity of involved cells against relevant molecular physiological regulators. The cells resistant to hormonal influences have no visible distinctions from the susceptible ones. In contrast, under conventional light microscope cancer cells look like abnormal versions of cells which compose the tissue of its origin. Light microscopy cannot perform an easy task to identify the site of a malignancy origin [5]. Thus the list of cancer uniqueness is far shorter than those one of its ordinariness.

### (c) Genetic immunity against molecular physiological regulators

Human body is provided with a physiological system that maintains its structures and functions within their genetically predetermined locations, shapes, sizes and molecular performance. The system acts through the molecular mediation i.e. by means of hormones and other molecular physiological agents. For instance, a dysfunction can be conditioned by deficiency of relevant hormone production. The same result can be achieved by mutant modifications of both the hormone and its receptor, which forms an incongruence between the co-actors, i.e. constitutional immunity against hormone influence [6-8].

The blocking effect of mutant modification of either hormones or their receptors leads to many pathological processes including obesity [9]. The immunity of cells to insulin is a major determinant of the decline of glucose tolerance. Similarly, non-insulin-dependent diabetes mellitus is characterized by pathological hyperglycemia in the presence of higher-than normal levels of plasma-insulin. Likewise, a pathogenic decrease in cell sensitivity to vitamin D3 determines the familiar forms of rachitic, and the immunity of cells to androgens causes the phenomenon of testicular feminization. Analogous resistance of cells to corticosteroids determines the pathogenesis of Cushing's disease [9]. The grade of the cells immunity to thyroid hormone determines the range of relevant disturbances. This resistance is an inherited inability to respond appropriately to the T3 hormone linked to mutations in the thyroid hormone receptor (TR)-beta [10].

The principles of cell immunity to physiological agents are analogous to those ones in constitutional (genetic) immunity to infections [4]. In a case of cancer the work of this precise and powerful regulatory system becomes also disturbed specifically. The mutual exposure, analysis and evolutionary comprehension of a set of relevant immunological data allowed us to put forward a new idea about molecular pathogenesis of cancer.

### (d) The proposed molecular pathogenesis of cancer

Humans are extraordinarily diverse in their manifestations of cancer. Affected people have many individual differences in manifestations of their cancer as well as in the grade of its expression. The shape, location, size and rate of cancer progression may be different in different individuals or in different organs as well. With the exception of impossible total affection on almost all the body that is only observed at leukemia, multiple myeloma, and lymphoma, several areas of restricted, focal, cancer locations exist in most other kinds of cancer. Cancer is presented by focal affections of individually different grades and locations [2]. The focalization of affections is the principal feature of any disease including cancer.

One can suppose that within any body affected by cancer there are initially two co-existing clones of homogenous cells with opposite predisposition to the development of the disease. The potentially cancerous clone exists in a form of distantly separated populations and their initial sizes are different but very small. They are dispersed around the body either stochastically or in a manner is not yet understood. One should suppose that their dispersion around the body has been performed before postnatal ontogeny [11]. Accordingly, the clone is not eliminated by mechanisms of adaptive immunity performed by lymphatic system. Initial subpopulations of a potentially cancerous clone are dispersed around the body like congenital small dark spots on human skin known as moles, the melanocytic nevuses, that exist in a form of benign neoplasm but may be at a higher risk for melanoma [12].

Different locations of potentially cancerous clone begin to be visually detectable in different times after initiation of their malignant growth that allow for the supposition of differences in their initially smallest sizes. The differences in such cancer cell masses and their dislocation around the body predestine individual diversity in the course and severity of cancer when the disease will begin its development.

Most cells of a cancer clone are mainly located in the organ or tissue of their belongingness. Other subpopulations of the same clone are located distantly. Some patients may have a cancer whose site of origin is hidden and never identified [5]. Various organs can serve the places for distant parts of a cancer to occur (the lungs, liver, brain, bones et al). The populations of malignant cells that formed leukemia, multiple myeloma, and lymphoma are usually not localized but dispersed around the affected body like the normal cells of the same tissue and relevant cancer cells may be found in the blood, several lymph nodes, or other parts of the body such as the liver and/or bones [2].

All distant locations of a cancer consist of the cells that are like those in the organ or tissue of its origin. This feature is paradigmatically considered as a result of metastasis [2]. In contrast to the paradigm it can be considered as a result of a process that still has yet to be understood, for instance either of the process of innate translocation or a transfer of a chromosomal segment to a new position, when a part of a chromosome is transferred to another chromosome especially on a non-homologous chromosome. Translocation can result in serious congenital disorders like sarcomas, leukemia, dysplasia et al.

For many initial decades of individual life the potentially cancerous cells exist in the body in full concordance with general rules of the regulation of cell reproduction. At a relevant time of individual age (mainly after 40 years of life), probably according to specific program of this cell population's ontogenesis, an unknown event induces their malignant development. Before or at the time the induction began the cells may be constitutionally resistant to normal regulators of both cell reproduction and the growth of cell populations inside. The existence of its constitutional immunity against relevant physiological regulators should be considered as the obligate prerequisite to malignity.

### (e) The specificity of genetic predilection to cancer

Although it is now an obvious truism that a person's genetic makeup has a principal influence on the development of cancer, the special characteristics of the genome that determine the resistance of cancer cells and focal distribution of cancer around the body are unknown. In contrast, the origin of analogous features of infectious diseases and many noninfectious ones as well as the role of constitutional immunity in their pathogenesis have been deciphered recently by many investigators [4].

The existence of genetic predilection to cancer is a most important source of doubts in the reality of metastasis. The predilection is predetermined by a set of genetic factors associated mainly with genetic admixture and with genesis of aging [13]. Genetic admixture (also called xenogamy, outbreeding, cross-fertilization, crossbreeding) refers to reproductive union of genetically dissimilar or unrelated organisms within the same species that resulted inevitably in the offspring's heterozygosity of various kinds. The states of heterozygosity are responsible for the origin, manifestations, course and severity of most diseases, both infectious and non-infectious ones [4]. Increased incidence of most diseases depends on the intensity of the population's genetic admixture. For instance, the number of obese people is increased in the territories with ethnically-mixed populations [9].

The role of xenogamy in the origin, manifestations and course of malignant diseases is evidenced by a plethora of epidemiological and clinical observations and investigations. African Americans are more likely to die from cancer then any other racial or ethnic population. In contrast, Hispanics, Asian Americans and Pacific Islanders have lower incidence rates than whites for the most common cancers. The frequency of colorectal cancer varies around the world. It is common in the Western world and is rare in Asia and Africa [2]. The cancerous insertion in the genome of humankind could happen on the yearly stages of anthropogenesis as a result of genetic admixture. This highly pathogenic insertion has not been eliminated by natural selection probably because it begins to function at the end of reproductive age.

Although only one cancerous clone exists usually in an affected body, the benigness of a number of such clones is documented too. Approximately one third of cancer survivors aged > 60 years were diagnosed more than once with another cancer. Possibly, this is associated with the phenomenon of intra-individual diversity in the progression of senescence [13]. In a population of a developed country with high survival rates multiple cancers comprise two or more primary cancers occurring in an individual that originate in a primary site or tissue and are neither an extension, nor a recurrence or metastasis [14]. Cancer patients have a 20% higher risk of new primary cancer compared with the general population. As the number of cancer survivors and of older people increases, occurrence of multiple primary cancers is also likely to increase [14-18]. Such observations induce the idea about possible existence in a body of few potentially cancerous clones.

## Discussion

The mutual exposure, analysis and evolutionary comprehension of epidemiological, clinical, immunological, genetic and experimental data concerning principal characteristics of cancer showed that many of them are also common to those of other kinds of diseases. Cancer is determined by a constitutional immune incongruence between relevant molecular cytoecological agents and their molecular targets. These features of individual molecular constitution exist in a population as a result of genetic admixture between people evolved in ecologically-distinct environments and have got differences in their molecular constitution on the level of cytoecological regulators of cell growth, development and differentiation.

Individual and intra-individual diversity in the cancer course, including the manifestations and severity of specific affections, their sizes and focal disposition around the body, could be created by inter-ethnic mating of persons with incongruent regulator-receptor systems. Thus, according to the hypothesis formulated and examined above, the genesis of cancer and its spread in human population are associated with both evolutionary formation of inter-ethnic differences and genetic admixture between genetically different persons.

In these circumstances, we should remember the commandment of Abraham: "… you will not take a wife…from the daughters of the Ca'naan'ites in among whom "… we are dwelling? [19]. The refusal of genetic admixture could be most effective way to prevent the growth of cancer incidence. What are the tests for the existence of cancerous genetic admixture that should be elaborated today and used tomorrow? The new notion on cancer origin, immuno-pathogenesis and epidemic spread provides a framework for understanding the epidemic nature of cancer and its rising incidence both in the developed world and in developing countries. It forces also to reassess the means and methods of cancer healing. What is more, it accents the possibility of genetic methods for the prevention of epidemic spread of the disease.
